# The Development of the Recovery Assessments by Phone Points (RAPP): A Mobile Phone App for Postoperative Recovery Monitoring and Assessment

**DOI:** 10.2196/mhealth.4649

**Published:** 2015-09-11

**Authors:** Maria Jaensson, Karuna Dahlberg, Mats Eriksson, Åke Grönlund, Ulrica Nilsson

**Affiliations:** ^1^ Faculty of Medicine and Health School of Health and Medicine Sciences Örebro Sweden; ^2^ School of Business Örebro University Örebro Sweden

**Keywords:** cellular phone, postoperative recovery, day care

## Abstract

**Background:**

In Sweden, day surgery is performed in almost 2 million patients per year. Patient satisfaction is closely related to potential adverse events during the recovery process. A way to empower patients and give them the opportunity to affect care delivery is to let them evaluate their recovery process. The most common evaluation method is a follow-up telephone call by a nurse one or two days after surgery. In recent years, mHealth apps have been used to evaluate the nurse-patient relationship for self-management in chronic diseases or to evaluate pain after surgery. To the best of our knowledge, no previous research has explored the recovery process after day surgery via mobile phone in a Swedish cohort.

**Objective:**

The objective of the study is to describe the process of developing a mobile phone app using a Swedish Web-based Quality of Recovery (SwQoR) questionnaire to evaluate postoperative recovery after day surgery.

**Methods:**

The development process included five steps: (1) setting up an interdisciplinary task force, (2) evaluating the potential needs of app users, (3) developing the Swedish Web version of a QoR questionnaire, (4) constructing a mobile phone app, and (5) evaluating the interface and design by staff working in a day-surgery department and patients undergoing day surgery. A task force including specialists in information and communication technology, eHealth, and nursing care worked closely together to develop a Web-based app. Modifications to the QoR questionnaire were inspired by instruments used in the field of recovery for both children and adults. The Web-based app, Recovery Assessment by Phone Points (RAPP) consists of two parts: (1) a mobile app installed on the patient’s private mobile phone, and (2) an administrator interface for the researchers.

**Results:**

The final version of the SwQoR questionnaire, which includes 31 items, was successfully installed in RAPP. The interface and the design were evaluated by asking for user opinions about the design and usefulness of the app with 10 day surgery patients. Some minor adjustments were made concerning text size and screen color.

**Conclusions:**

Taking advantage of joint expertise, a useable Web-based app adaptable to different technical platforms was constructed. In addition, the SwQoR was successfully transferred into digital format for use on mobile phones.

## Introduction

### Day Surgery

In Sweden, almost 2 million day surgeries are performed in adults each year [1,2]. The literature has different definitions of day surgery, varying from the patient going through surgery and staying in a patient hotel overnight, to same-day admission and discharge [3,4]. There are few patient-related contraindications to day surgery, but social and medical factors are both assessed in order to select suitable patients [3]. The advantages for patients undergoing day surgery include a lower risk of hospital infections, earlier mobilization, and the convenience of recovering at home. For health care providers, day surgery is cost-effective and spares beds for other surgical cases [3]. From the perspective of safe and effective day surgery, anesthesia must minimize the postoperative discomfort of patients. Working with the goal of rapid recovery, the induction agents must have rapid and smooth onset [3], the airway needs to be carefully managed [5], and the risk for postoperative nausea and vomiting (PONV) and postoperative pain needs to be addressed before and after surgery [3,6]. A study reported that 82% of patients are discharged <270 minutes after surgery. Delayed discharge was mainly due to adverse events, such as PONV and pain [6]. According to a large survey involving more than 12,000 patients, the most common complaints after surgery with general anesthesia are PONV, sore throat, and hoarseness [7]. Other reported adverse events are dental damage, headache, urine retention, and confusion [8]. Women also seem to be more likely to experience adverse events than men [8]. Though objective symptoms have traditionally been monitored as an integrated part of care and treatment [1], patients’ subjective descriptions (patient-reported outcomes measures) have come to be considered a fundamental element of measure and follow-up [9,10]. During the first two weeks of recovery, many patients experience symptoms requiring unplanned health care contacts, phone calls, or outpatient clinic visits [4] and, in North America alone, these unexpected visits and readmissions to hospitals cost billions of dollars annually [11].

### Follow-Up Assessment Studies

Studies show that patient satisfaction is directly related to their experience with adverse events related to anesthesia and surgery [7,12]. However, according to a previous survey conducted in Sweden, not all units performing day surgery have implemented routines for follow-up assessment [4]. Also, several studies have reported that the most common method of follow-up is a phone call from a nurse 1-2 days after surgery [1,4]. This procedure can be seen as time consuming for personnel and not cost effective. A face-to-face meeting with a nurse anesthetist or an anesthesiologist would be desirable for follow-up, but as this is difficult to achieve, an alternative is to use technological solutions [11]. As a subcategory of eHealth, the new concept of mHealth refers to using mobile phones in health care [13]. Patient use of the Internet via mobile phones, such as self-management by means of text messaging persons with diabetes or asthma [14,15], providing prevention information for breast cancer [16], and soliciting experiences with pain after surgery [17], has been reported in different chronic diseases to improve the nurse-patient relationship. However, limited knowledge is available about how information and communication technology is perceived in the peri-operative context [11]. To the best of our knowledge, a Swedish cohort of day-surgery patients has not yet been explored. The present study describes the process of developing a mobile phone app using a Swedish Web-based Quality of Recovery (SwQoR) questionnaire for evaluating postoperative recovery after day surgery.

## Methods

### Development Process

The development process included the following steps: (1) setting up an interdisciplinary team, (2) evaluating the potential needs of app users, (3) developing the Swedish Web version of a QoR questionnaire, (4) constructing a mobile app, and (5) evaluation of the interface and design of the app by staff working at a day-surgery department and patients undergoing day surgery.

### The Interdisciplinary Research Team

Interdisciplinary research involves the translation of scientific knowledge between members of the research team [18]. In this specific project, it was important to include researchers with broad expertise and perspectives that would enrich the research team. This includes the project leader who is a professor and head of the “Perioperative nursing” research environment, with broad experience in intra and postoperative care, both as a nurse anesthetist and as a researcher; an associate professor in pediatric nursing, with research focus on measuring health conditions in children and adolescents; a professor in informatics, who is experienced in implementing information systems in organizations and evaluating their effects on processes and users, with a special focus on public sector organizations in Sweden and internationally; and a professor in information systems development, and informatics, with specialization in information security and privacy. The team also includes an associate professor and anesthetist, with a broad experience in anesthesia and postoperative care, both as a clinician and a researcher, and who has knowledge on postoperative cognitive dysfunction; a professor and psychologist, with broad experience and knowledge in cognitive impairment and cognitive aspects; and a senior lecturer with a PhD in nursing and knowledge in nursing informatics. Finally, the team includes a health economist (PhD), a postdoctoral researcher (in nursing) who is also a nurse anesthetist, and a doctoral student (in nursing), with experience in day surgery and postoperative care.

Prior to the first meeting, all members in the group read the same articles describing mHealth [19]. To facilitate cooperation in the team, the researchers started by discussing a joint framework with clear goals. The next step was to define the focus of the app (Table 1).

**Table 1:** Focus of the app from the perspectives of the health care organization and the patient.

Health care organization	Patient
To get reports back from the patients	Provide personalized feedback to the health care about the recovery process
To support the management of the individual patient in follow-up contacts by a health professional	A feeling of being cared for
	A sense of empowerment
To reduce serious recovery problems associated with suffering and costs	
To learn more about postoperative reactions and recovery and to improve surgical and anesthetic procedures in the long-term	
Being easy to understand for nurses and medical doctors in the health care system	Being easy to understand for patients in the health care system
Reduce unplanned or unnecessary health care contacts	Reduce unplanned or unnecessary health care contacts

### Evaluating the Potential Needs of App Users

In order to determine the potential needs of users of the app, the researchers reviewed the literature and brainstormed during a workshop. The members shared their own personal experiences in postoperative recovery as both patients and researchers in the field of anesthesia and postoperative care. In order to make the app user friendly, the research team established that it would be important to use the patients’ own mobile phone [17], rather than a specified mobile phone for the research project [11]. A review of the literature showed that mobile phone technology needs to be user friendly, easy to navigate, and not show a large amount of text on the screen [20]. Also, the use of a push function can encourage individuals to leave a response at a given time [17]. Finally, it was hypothesized that users being able to compare their recovery to a sample of other patients would give them a sense of empowerment.

### Swedish Web Version of a Quality of Recovery Questionnaire

Myles et al [12] developed the instrument QoR-40, which has been adapted to a Swedish day surgery context for adults as the QoR-24 [21]. A meta-analysis including 18 studies (3459 patients) concluded that the QoR-40 has excellent validity, reliability, responsiveness, and clinical utility for a broad range of patient populations [2]. The Swedish version of QoR-24 [21], together with inspiration from the newly developed questionnaire Postoperative Recovery in Children (PRiC; personal communication with Ulrica Nilsson, April 27, 2015), the Postoperative Recovery Profile [22], the Post-discharge Surgical Recovery scale [23], and Nilsson and Idvall´s study [24], contributed to the final version of the SwQoR, which includes 31 items (Table 2). The items applied from QoR-40 [11] and QoR-24 [21] were originally scored on a 5-point scale (for positive items, 1=none of the time to 5=all of the time; for negative items the scoring was reversed). In line with Stark et al [25], the scaling properties and options of obtaining verbal numerical responses would be easy to understand for the patients. We changed the format of the pain rating scale used in clinics to a horizontal visual analog scale from 0 (“none of the time”) to 10 (“all of the time”). At the end of the questionnaire, the patients are asked if they want to be contacted by a nurse (response alternative YES or NO). If the answer is YES, a nurse at the day-surgery department contacts the patient and offers further information and assistance.

**Table 2:** The revision and rationale for items in the SwQoR.

QoR-24	Revision/rationale	SwQoR
Able to breathe easy	-	Able to breathe easy
Sleeping well	-	Sleeping well
Being able to enjoy food	-	Being able to enjoy food
Feeling rested	-	Feeling rested
Having a general feeling of well-being	-	Having a general feeling of well-being
Feeling in control	-	Feeling in control
Pain in the surgical wound	-	Pain in the surgical wound
Feeling relaxed	-	Feeling relaxed
Speaking normally	-	Speaking normally
Able to brush teeth	Merged into one item and linguistic revision	Able to look after personal hygiene
Able to look after own appetence		
Able to write	Linguistic revision	Able to write as usual
Able to return to work	Linguistic revision	Able to return to work or usual duties about the home
Nausea	Merged into one item	Nausea and vomiting Nausea or vomiting
Vomiting		
Feeling restless	-	Feeling restless
Shivering or twitching	-	Shivering or twitching
Feeling too cold	-	Feeling too cold
Dizziness	-	Dizziness
Nightmares	-	Nightmares
Anxiety	-	Anxiety
Depressed	-	Depressed
Feeling lonely	-	Feeling lonely
Difficulties getting to sleep	-	Difficulties getting to sleep
	[24]	Headache
	[24]	Muscle pain
	[24]	Back pain
	[24]	Sore throat
	[22]	Difficulties concentrating
	[22]^a^	Trouble urinating
Difficulties defecating	[22,23]^a^and divided into two items	Feeling constipated
		Diarrhea

^a^From PRiC, personal communication with Ulrica Nilsson, 20150427

### Constructing a Mobile App

Our goals were that the app should be easy to use, be safe and secure, and allow aggregation of data to a study database. A wishing list of app functions, interface, and design were established and presented to the commissioned software company, which developed the Web-based app Recovery Assessed by Phone Points (RAPP) in close collaboration with the interdisciplinary team.

The technical solution consisted of two parts, a mobile app for patients and a Web-based administrator interface for the researchers. A patient interacts with the mobile app to report his or her postoperative recovery. In the development and testing phases, the app was designed in HTML5 (the most recent version of the markup language used for structuring and presenting content for the World Wide Web) and JavaScript to largely mimic a native mobile app. The Web-based technical solution was a mobile app platform that made it easier to implement the solution regardless of the end user’s technical equipment. Thus, it is possible to use RAPP regardless of the type of mobile phone being used.

In the development phase, none of the data imputed by patients were collected. Study-specific log-in codes were set up and used in connection with the installation of RAPP on the participants’ mobile phone.

### Evaluating Interface and Design

A difference between answering a questionnaire on the small screen on a mobile phone and answering on a paper is that one item at a time is shown on the screen versus multiple items on a paper page [26]. The app’s interface and design were evaluated asking for user opinions about the design and usefulness of the app. There were ten day-surgery patients that were recruited from two day-surgery departments in Sweden. Patients who were included brought their own mobile phone to the day-surgery department at one of two specific days when the testing took place. No one who was asked about participating in the testing declined. The app was installed on the patient´s own mobile phone by the researcher. Instructions about the SwQoR and how to navigate the app were given. The questions asked to the patients were: (1) “What is your opinion about the layout?”, (2) “Can you describe any obstacles when using the app?”, (3) “What is your overall opinion about the app?”, and (4) “Do you think that this would be a useful method to use after ambulatory surgery?”

A member of the research team wrote down all responses as field notes. Staff working at one of the day-surgery departments also had the opportunity to provide feedback on the device’s interface. The included staff members were nurses (n=10), surgeons (n=5), and anesthesiologists (n=2), all with experience from working in a day-care setting. This testing was carried out in connection with a lecture about postoperative recovery, in which also the app (RAPP) was demonstrated. The staff was invited to give their opinion about the layout of the interface. There was one researcher that took field notes.

### Ethical Considerations

As the study did not collect or handle any sensitive personal data, ethical approval was not required according to the Swedish Act concerning the Ethical Review of Research Involving Humans (SFS 2003: 460) [27]. Nevertheless, the study followed standard research ethical principles, and the project did not collect or handle any sensitive personal data. The participants were given written or verbal information (depending on the clinical guidelines when contacting the patient before surgery) about the study, including the purpose and procedures and that participation was voluntary by the staff working at the day-care department. The participants were also asked to bring their mobile phones to the day-surgery department at the day of surgery. When arriving to the day-surgery department, members of the research team gave oral information about the testing. The patients were guaranteed that no personal data would be collected. After asking for user opinions about the design and usefulness of the app, the Web-based app was uninstalled from the patient’s mobile phone. No data from the SwQoR were collected for further analysis.

## Results

**Figure 1:**
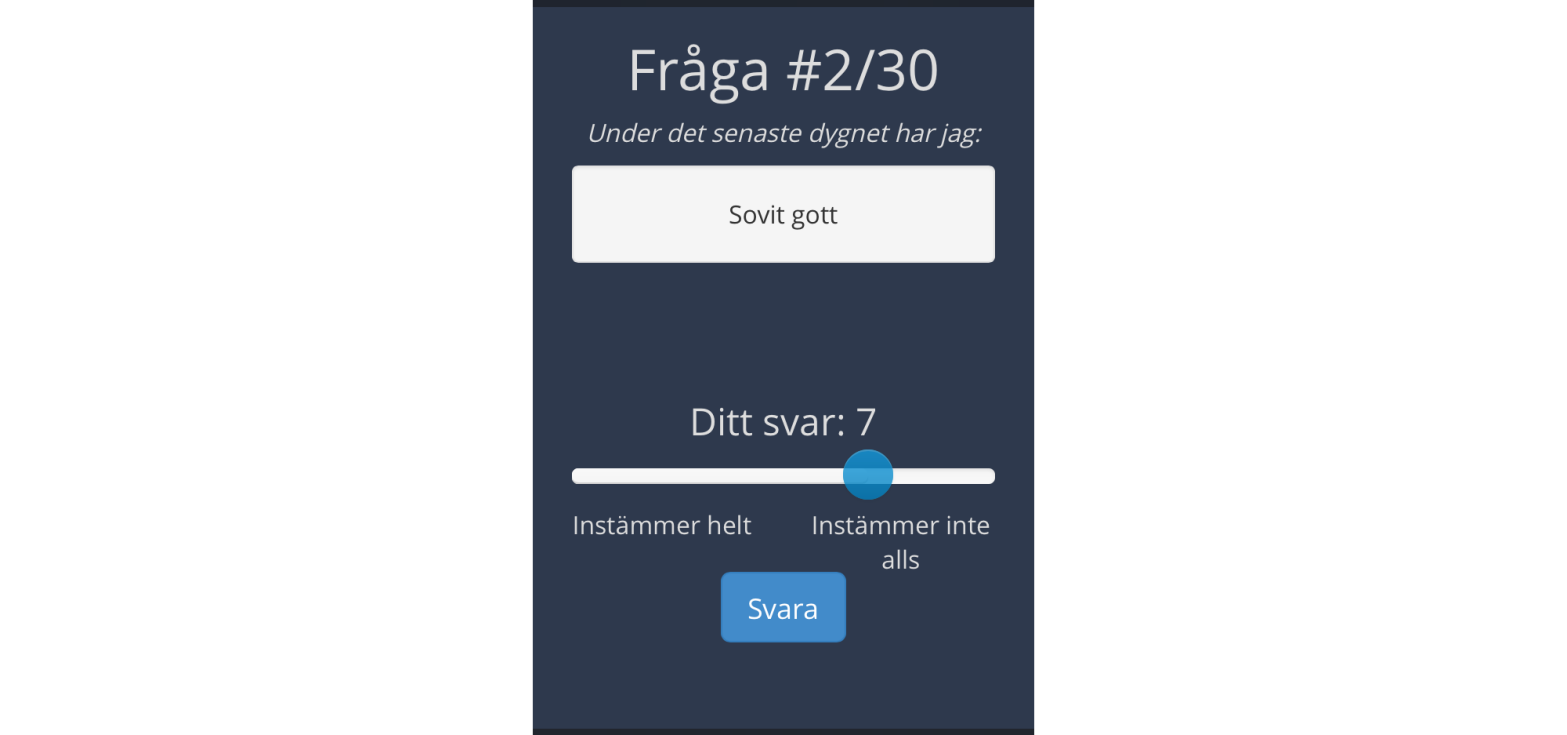
An example of the Recovery Assessed by Phone Points (RAPP) after the patients’ feedback, the background has a darker background and the text has been increased. (During the last 24 hours I have: Slept well, None of the time-All the time).

There were four patients that reported that the background color was an issue and three patients had comments about the text size. Regarding obstacles to use, five patients thought the scale to be confusing. There were two that found it impractical that they could not move back and forth between questions. When discussing the overall opinion of the app, three patients suggested that it would be easier if the dot on the visual analog scale (Figure 1 shows this) could be moved also by touching the line to choose score, instead of drawing the dot with the index finger. Overall, all ten patients expressed a positive attitude toward the method of evaluating postoperative recovery using an app.

The staff working in the day-care settings gave similar feedback. They commented about the text size, the background color, and several of the staff found the scale confusing. Overall, all staff were positive to RAPP. They found the questions in the SwQoR relevant, and they also confirmed the need for systematic follow-up in the recovery process.

**Figure 2:**
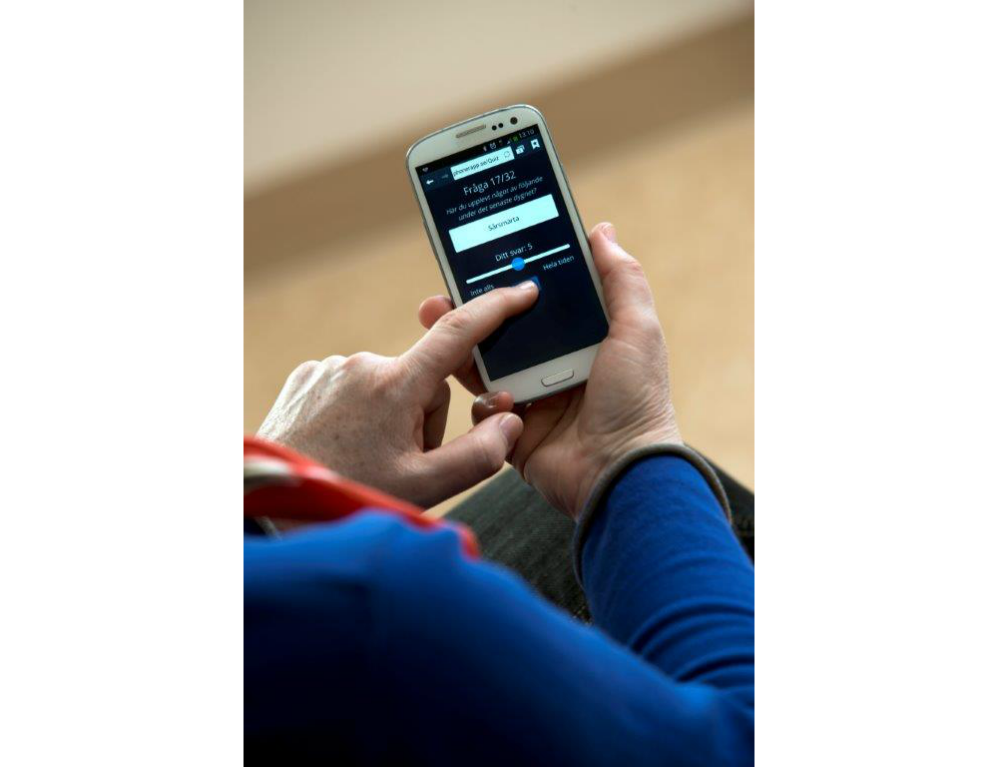
The patient can move the dot simply by touching the line. (During the last 24 hours Have you had any of the following: pain, None of the time-All the time) © Ulla-Carin Ekblom.

Patient and staff feedback led to several changes in the app. The text background was changed to a darker color (Figure 1). The size of the text was also increased and the scale was clarified. At first, the visual analog scale was rated 0 “all of the time” and 10 “none of the time” for positive items and the opposite for negative items, which confused both patients and staff. Therefore, the scale was changed to 0 “none of the time” and 10 “all of the time” for all items whether the item was negative or positive. Regarding the dot along the visual analog scale, the opportunity to choose score by touching the line was added (Figure 2 shows this). The dot on the visual analog scale line was also programmed to go back to neutral, 5, each time a new question was shown on the screen to make it clearer that a new question was to be answered.

During the testing, it became clear that not all of the text was visible on the screen of some older mobile phone models with small screens. Thus, the app was reprogrammed so that it would also fit smaller screens. Overall, the app was considered easy to use, to understand, and to navigate by both patients and personnel.

## Discussion

### Principal Findings

This project is unique in its intention to develop a mobile phone app that will be used with the patients´ own mobile phones in the peri-operative context. To our knowledge, there are no published papers with focus on the development process of an app for evaluating postoperative recovery. However, in others areas, there are some newly published articles [28,29]. Therefore, this paper demonstrates the process of establishing an interdisciplinary research team, which together developed a useable app in regard to interface, design, and utility, for which testing was done with both patients and personnel. To the best of our knowledge, no systematic assessments of patients’ postoperative recovery, paper-based, Web-based, or mobile phone-based, are yet available. The majority of previously published national and international studies have developed mobile apps for use on devices provided by the research projects. For example, to study the use of a mobile app to monitor postoperative recovery, Semple et al [11] gave the patients either a mobile phone or a tablet with the app installed on the device prior to discharge. This uniqueness of the present study is a strength with regard to implementation, as it would be difficult to convince the health care system to adopt the costs for providing all patients with devices for self-reporting.

A new patient safety law (The Swedish Code of Statutes, SFS 2014:821) was implemented in January 2015. This law gives patients even more power to affect their care. Notably, patient participation is a core element in patient-centered care [30], and it is crucial to involve patients and evaluate the care provided, in this case anesthesia and postoperative care. A benefit of using the RAPP could be increased patient satisfaction, as well as improving peri- and postoperative care in specific groups.

The provision of health care can be evaluated using different methods, each with advantages and disadvantages. A paper questionnaire is easy to use, has low implementation cost, and needs little support to the patients, but disadvantages include costly delivery of the questionnaire and potential negative attitude of respondents toward answering a lot of questions [26]. The advantages of technical tools, such as mobile phones and tablets, include a reduction in missing data by requiring completion of the item and only allowing one item at a time on the screen so as to improve the response rate. Furthermore, a mobile phone is easy to carry around and frequently used by most people in everyday life [31]. The downside of a technical solution is that some individuals do not have private mobile phones, though this group is decreasing more and more. Moreover, the attitude and ability of the individual, for example, lack of interest or limited knowledge of mobile phone use, could be an obstacle [16].

A number of studies have compared electronic evaluation of patient-reported outcomes (ePRO) and paper and pencil administration and shown an advantage for ePRO [32,33]. However, there seems to be a lack of knowledge concerning the use of a mobile phone app instead of pen and paper to collect patient-reported outcomes in a peri-operative context. For further development of RAPP and of the SwQoR, there is an ongoing study with the aim of exploring the difference between the two methods’ ability to assess patient-reported outcomes as suggested by Coons et al [32]. This study will show if there is equivalence between the two questionnaire delivery modes (paper vs app). In the mentioned pilot study, patients undergoing day-care surgery evaluated the acceptability and feasibility of the app. In the near future, a multicenter randomized controlled study (n=1000) with a primary outcome of cost effectiveness and secondary outcomes of postoperative recovery, QoL, overall health, and health literacy will be performed by our research team. Future studies include also qualitative research evaluating the patients’ experience of the intervention and the staffs’ experience of the implementation.

According to literature, mobile features used in other studies are, for example, text messages, pedometers for physical activity, or video, voice, or multimedia messages [14]. The RAPP is designed to solve some problems, not all. The problems we set out to solve are well defined, and hence, so can the solution be. Some functionality, such as personalized feedback, is extremely difficult to design (as there are many possible situations and multiple factors involved) and do more harm than good if not accurate enough. Therefore, this project aims at making improvements to some problems to which there are credible and robust solutions. Solving them is a big step forward. In the future, further functionality may be added. Patient requirements for other functionalities as well as opportunities to actually implement such will be further analyzed in our upcoming studies. Our research team is planning on developing RAPP in an ongoing process, and, thereby including the patients by continuing to evaluate the patients´ need for support in the postoperative process.

Our projects overall aims are to integrate society’s need for quality auditing and assurance in health care with the patients´ need for safe and reliable information and communication regarding their postoperative recovery. We believe that the project will increase patients’ self-care. Using systematic follow-up remote symptom monitoring during postoperative recovery enables evaluations and comparisons of the usefulness and cost-effectiveness of different technical approaches to care, drug treatment, care activities, and competence development. It is also hoped that the use of systematic follow-up will help guide improvements in areas of anesthesia and postoperative care among patients who currently have low-quality postoperative recovery.

### Limitations

Having members from different disciplines work together is a way of avoiding fragmented research [18]; this is one of the strengths of this research team. The development and implementation of a Web-based app can overcome some barriers, which were discussed by the members of the team. A barrier includes the type of device, as some people may prefer their tablet or computer to a mobile phone. Another barrier may be the small screen size, which could be difficult to read or handle. A slow Internet connection or slow app loading time could be a problem for users at home, the app will need to be usable on both mobile phones and tablets, and both the app and the data it transfers should be small in size to minimize network load and memory usage.

The respondents are also asked to give responses about their recovery after anesthesia and surgery. The patients may be affected by the residuals of anesthesia and miss responding within a specific time frame [17]. In order to improve the response rate in the pilot study, a reminder in the form of a text message will be sent to the respondent each day.

Another factor that could affect the use of health care information technology is staff attitudes toward technology and perhaps the fear of dehumanizing care [34]. To counteract this risk, the members of the research team included both a nurse anesthetist, a nurse with experience in the post anesthesia care unit, and an anesthesiologist. All team members were involved in the app development process together with the software company. This approach may improve attitudes toward and the usability for nurses and physicians in the peri-operative context [34].

### Conclusions

With joint expertise, a useable Web-based app, RAPP, adaptable to different technical platforms was developed and tested for understandability and user-friendliness. The SwQoR has also successfully been transferred into digital format for use on mobile phones.

## Abbreviations

ePRO: evaluation of patient-reported outcomes
PONV: postoperative nausea and vomiting
PRiC: Postoperative Recovery in Children
RAPP: Recovery Assessed by Phone Points
